# Integrative analysis of differentially expressed microRNAs of pulmonary alveolar macrophages from piglets during H1N1 swine influenza A virus infection

**DOI:** 10.1038/srep08167

**Published:** 2015-02-02

**Authors:** Pengfei Jiang, Na Zhou, Xinyu Chen, Xing Zhao, Dengyun Li, Fen Wang, Lijun Bi, Deli Zhang

**Affiliations:** 1 College of Veterinary Medicine, Northwest A&F University, Yangling, Xi'an City, Shaanxi, 712100, P. R. China; 2 Department of Microbiology & Immunology, School of Basic Medical Sciences, Wenzhou Medical University, Wenzhou, Zhejiang, 325035, P. R. China; 3 State Key Laboratory of Biology Macromolecule, Institute of Biophysics, Chinese Academy of Sciences, 100101, Beijing, P. R. China

## Abstract

H1N1 swine influenza A virus (H1N1 SwIV) is one key subtype of influenza viruses with pandemic potential. MicroRNAs (miRNAs) are endogenous small RNA molecules that regulate gene expression. MiRNAs relevant with H1N1 SwIV have rarely been reported. To understand the biological functions of miRNAs during H1N1 SwIV infection, this study profiled differentially expressed (DE) miRNAs in pulmonary alveolar macrophages from piglets during the H1N1 SwIV infection using a deep sequencing approach, which was validated by quantitative real-time PCR. Compared to control group, 70 and 16 DE miRNAs were respectively identified on post-infection day (PID) 4 and PID 7. 56 DE miRNAs were identified between PID 4 and PID 7. Our results suggest that most host miRNAs are down-regulated to defend the H1N1 SwIV infection during the acute phase of swine influenza whereas their expression levels gradually return to normal during the recovery phase to avoid the occurrence of too severe porcine lung damage. In addition, targets of DE miRNAs were also obtained, for which bioinformatics analyses were performed. Our results would be useful for investigating the functions and regulatory mechanisms of miRNAs in human influenza because pig serves as an excellent animal model to study the pathogenesis of human influenza.

Influenza viruses are enveloped, negative-strand RNA viruses that are transmitted through contact with infected individuals or contaminated items, and through inhalation of aerosols, leading to seasonal outbreaks of acute respiratory tract infection. Although yearly vaccination can provide some measure of protection, rapid mutation can yield emerging variants of influenza with the potential to cause pandemic infections^1^,^2^.

H1N1 swine influenza A virus (H1N1 SwIV) is one key subtype of influenza viruses, which circulates in pigs worldwide causing swine influenza characterized by fever, anorexia, tachypnea, dyspnea and coughing^3^,^4^. In March 2009, a novel swine-origin H1N1 influenza A virus containing gene segments from swine, human, and avian influenza viruses circulated in humans and raised severe concerns about pandemic developments^5^, which indicates the significant role of H1N1-subtype influenza A virus in the evolution of new viruses with pandemic potential^6^.

Swine influenza has the characteristics of lasting for a short period and quick recovery. Lung is the major target organ for H1N1 SwIV infection because its replication is mainly restricted to epithelial cells in the respiratory tract^7^. However, there is no report whether pulmonary alveolar macrophages (PAMs) can be infected by H1N1 SwIV. As a short-lasting disease, swine influenza manifests itself with an incubation period of 1-3 days, then the recovery phase follows, which is limited to 6 or 7 days after infection^8^. This suggests that a large number of anti-viral molecules may play a central role in the infection course, which has been partly proved by previous studies. During the acute phase of the disease, H1N1 SwIV induces an overwhelming and simultaneous pro-inflammatory cytokines in the lungs of infected pigs such as Th1, Th2, Th3, IFN-alpha, tumor necrosis factor-alpha, interleukins, and so on^5^,^9^,^10^. Among these cytokines, several have been demonstrated to be related with anti-viral functions or tissue damage at the acute stage of SwIV infection.

MicroRNAs (miRNAs) are endogenous, non-coding 21- to 23-nucleotide small RNA molecules that regulate gene expression by binding to the untranslated region of target mRNAs, leading to their translational inhibition or degradation^11^. Many studies have indicated miRNAs are attractive candidates as upstream regulators, because miRNAs can post-transcriptionally regulate the entire set of genes^12^. It is well known that many miRNAs are expressed after a specific virus infection or at a specific developmental stage. Moreover, in the analysis of the gene regulatory mechanisms, attention has been given to key factors in the clinical course and pathology of the disease, particularly when the host is infected with zoonosis, like SwIV. Therefore, identifying specific miRNAs is the first step to understand the biological functions of miRNAs during H1N1 SwIV infection.

The clinical manifestations and pathogenesis of influenza in pigs closely resemble those observed in humans^5^. Like humans, pigs are also outbred species, and they are physiologically, anatomically, and immunologically similar to humans^13^,^14^. In contrast to the mouse lung, the porcine lung has marked similarities to its human counterpart in terms of its tracheobronchial tree structure, lung physiology, airway morphology, abundance of airway submucosal glands, and patterns of glycoprotein synthesis^15^,^16^,^17^. Furthermore, the cytokine responses in bronchoalveolar lavage fluid from SwIV-infected piglets are also identical to those observed for nasal lavage fluids of experimentally infected humans^18^. Recently, several studies have taken porcine genes such as BCL-G^19^, p58^IPK^^20^, and JAB1^21^ as molecular models to study human diseases. These reports indicate that the pig can serve as an excellent animal model to study the pathogenesis of human influenza.

As mentioned above, many proteins, especially some cytokines, were found to be related to H1N1 SwIV. However, miRNAs related to H1N1 SwIV have rarely been reported. To obtain sufficient information on host response to H1N1 SwIV infection, the present study focused on differentially expressed (DE) miRNAs in pulmonary alveolar macrophages (PAMs) from piglets during the H1N1 SwIV infection. The comparison of H1N1 SwIV-infected and control PAMs indicated that 70 and 16 known miRNAs were differentially expressed respectively on post-infection day (PID) 4 and PID 7. The comparison of H1N1 SwIV-infected PAMs between PID 4 and PID 7 indicated that 56 known miRNAs were differentially expressed. As a result, these data would enable us to better understand the underlying pathogenesis of H1N1 SwIV infection in piglets.

## Methods

### Ethics Statement

Our study had been approved by Animal Care and Use Committee of Shaanxi Province, China. All animal procedures were performed according to guidelines developed by the China Council on Animal Care and protocol approved by Animal Care and Use Committee of Shaanxi Province, China.

### Piglets and virus infection

A litter of six conventionally-reared, healthy 6-week-old, Yorkshire piglets was selected from a high-health commercial farm that has historically been free of all major pig diseases, such as H1N1, PRRSV, porcine circovirus type 2, classical swine fever virus, porcine parvovirus, pseudorabies virus, swine influenza virus and mycoplasma hyopneumoniae infections. All piglets were H1N1-seronegative determined by ELISA (HerdChek PRRS 2XR; IDEXX Laboratories) and absence of H1N1 tested by RT-PCR. Piglets were randomly assigned to three groups in the experiment and raised in isolated rooms. Four piglets were respectively inoculated with 10^5^ TCID_50_/ml H1N1 (A/Swine/Guangdong/LM/2004(H1N1))^22^ by tracheal injection (3 ml). Two uninfected negative control piglets were treated similarly with an identical volume of PBS. H1N1 SwIV-infected piglets were clinically examined daily and rectal body temperatures were recorded from PID 1 to 7. Viral reisolates were performed after these piglets were killed. The infected group showed positive while the control group was negative.

### Histopathological analysis

Two control piglets were euthanized on PID 0. Two infected piglets randomly chosen were euthanized on PID 4 and the rest two piglets were euthanized on PID 7. All piglets were euthanized by exsanguinations after intravenous administration of pentobarbital. Lung tissues from piglets in three groups were collected and the macroscopic lesions were estimated visually. Then, Lung tissues were fixed in 10% (w/v) buffered formaldehyde for 36 h, and embedded in paraffin according to standard laboratory procedures. Serial sections were cut 5 μm thick from each of the formalin-fixed, paraffin-embedded tissues and were processed for hematoxylin-eosin (HE)-staining according to standard protocols.

### RNA isolation and small RNA library construction

PAM samples were respectively collected from two control piglets on PID 0, two piglets on PID 4, and two piglets on PID 7 using lung lavage technique as previously described^23^. These PAM samples were observed by light microscopy to determine the purity in approximately 95% and then were immediately frozen in liquid nitrogen for RNA isolation.

Total RNA from PAM samples was extracted using the mirVana™ miRNA Isolation Kit (Ambion, Austin, TX) according to the manufacturer's instructions. RNA samples from two piglets on PID 0, PID 4 or PID 7 were respectively mixed as sample on PID 0, PID 4 or PID 7. The RNA quality of three samples was assessed using a BioAnalyzer 2100 (Agilent Technology, Santa Clara, CA). The purified RNA was quantified by determining the absorbance at 260 nm using a Nanodrop ND-1000 spectrophotometer (Infinigen Biotechnology Inc., City of Industry, CA). RT-PCR was used to determine the infection of H1N1 SwIV in PAM samples on PID 4 and PID 7. Exactly, PB2 gene was amplified with the following primer pair: forward, 5′-AATTACAACAAAGGCACCA-3′ and reverse, 5′-GCTTCCGTTTCATTACCA-3′. RNA samples were sequenced with Solexa/Illumina platform by BGI (Beijing Genome Institute at Shenzhen, China).

For small RNA library construction and deep sequencing, the 18–30 nt size range of RNA was enriched by polyacrylamide gel electrophoresis and then 20 µg of the purified small RNA from each sample was subject to DNA sequencing with an Illumina Genome Analyzer (Illumina, San Diego, CA) according to the manufacturer's instructions. In brief, proprietary adapters were then ligated to the 5′ and 3′ termini of these small RNAs, of which the ligated small RNAs were then used as templates for cDNA synthesis. The cDNA was amplified with 18 PCR cycles to produce libraries that were sequenced using Solexa's proprietary sequencing-by-synthesis method. The image files generated by the sequencer were then processed to produce digital-quality data. After masking of adaptor sequences and removal of contaminated reads, we got the clean reads of full-length small RNA sequences for further analysis. To confirm the quality of these sequencing data, we calculated the average quality score of sites and reads of each sample.

### Search for known miRNAs expressed in each sample

To identify known porcine miRNAs (known miRNAs refer to miRNAs that have been included in miRBase) expressed in each sample, the miRBase version 19.0 containing 306 mature porcine miRNAs (http://www.mirbase.org/) was downloaded and a BLASTN search was performed to align the unique sequence reads with porcine precursor miRNA sequences. The hits were considered a real match if there were a minimum of 18 nucleotides matched between the sequence read and the miRNA from the database.

### Identification and analysis of DE miRNAs

To identify the DE miRNAs between samples, the count of one known miRNA in each sample was normalized against the total counts of all known miRNAs in this sample. The normalization formula is described as follows: Normalized expression level = the count of one known miRNA/total counts of all known miRNAs × 10^6^. Then, the fold change of one miRNA between samples was calculated, and the following formula was used: Fold change = log 2 (treatment/control). Statistical analysis was performed and significance values were determined as previously described^24^. A p-value below 0.01 was considered as significant. Scatter plots were created to visually demonstrate the changing trends of known miRNA expression levels. Published references were searched to identify DE miRNAs that were conservative between pig and humans during the infection with H1N1 influenza A virus.

MiRNA expression pattern clustering was performed using miRNAs with significant expression variance. All DE miRNAs were clustered using the hierarchical cluster (Version 3.0) software and the results were visualized using the TreeView (Version 1.1.1) software.

### Quantitative real-time PCR (qRT-PCR) experiments

To validate the sequencing results, qRT-PCR experiments were conducted for 5 randomly selected miRNAs using the All-in-One^TM^ miRNA qRT-PCR detection system (GeneCopoeia, Rockville, USA). Briefly, total RNAs from three groups were prepared as mentioned above. Then, 100 ng of total RNA was used to conduct reverse transcription of miRNAs with the All-in-One^TM^ miRNA First-Strand cDNA synthesis kit (GeneCopoeia, Rockville, USA) as per the manufacturer's instructions. 2 μl of first-strand cDNA (diluted at 1:5) was used to conduct the qRT-PCR with the miRNA-specific forward primers and the universal adaptor PCR primer (provided by the qPCR kit) using the All-in-One^TM^ miRNA qPCR Kit as per the manufacturer's instructions. All reactions were run in triplicate, and porcine U6 snRNA was used as an endogenous reference. The ΔΔCt method was used to calculate the expression level differences of miRNAs between examined samples. The miRNA-specific forward primers and U6 snRNA primers were listed in Table S1.

### Identification of transcription factors (TFs) relevant with DE miRNAs

To identify TFs which regulate the expression of DE miRNAs, the data of interactions between TFs and miRNAs were downloaded from TransmiR database (http://202.38.126.151/hmdd/mirna/tf/). Then, DE miRNAs were aligned with these data to find the relevant TFs.

### Targets prediction for DE miRNAs

To predict targets of DE miRNAs, our strategy included three parts. For DE miRNAs whose sequences are absolutely consistent with that of human miRNAs with targets verified by experiments, targets of human miRNAs were downloaded from miRecords (http://mirecords.biolead.org/), miRTarBas (http://mirtarbase.mbc.nctu.edu.tw/index.html), or TarBase (http://diana.cslab.ece.ntua.gr/tarbase/) as targets of homologs in piglet. For DE miRNAs whose sequences are absolutely consistent with human miRNAs without targets verified by experiments, predicted targets of human miRNAs were downloaded from DIANA-MICROT (http://diana.pcbi.upenn.edu/cgi-bin/micro_t.cgi), MIRDB (http://www.mirdb.org/), or TargetScan (http://www.targetscan.org/) as targets of homologs in pig. For DE miRNAs whose sequences are different from that of human miRNAs, targets were predicted based on 3′UTR sequences of pig genome and sequences of pig miRNAs using software miRanda, PITA, or RNAhybird, respectively. Then, the intersected targets from these three softwares were used as the predicted targets.

### Functional annotation analyses of DE miRNA targets

To fully investigate the functions of the DE miRNA targets, we performed Gene Ontology (GO) enrichment and Kyoto Encyclopedia of Genes and Genomes (KEGG) analyses for the predicted miRNA targets using the david gene annotation tool (http://david.abcc.ncifcrf.gov/). Hypergeometric test and Benjamini & Hochberg false discovery rate were performed using the default parameters to adjust the p-value (p<0.05).

### Construction of protein-protein interaction (PPI) network regulated by DE miRNAs

Firstly, 33718 porcine protein sequences were downloaded from Uniprot database and saved as FASTA format. According to target genes encoding proteins and the same accession number for one protein in different databases, information of proteins regulated by DE miRNAs was obtained by mapping the predicted target genes to proteins. Then, target proteins were mapped to porcine PPI network containing 567586 pairs of PPI constructed by Wang et al.^25^. The PPI network regulated by DE miRNAs was visualized using the Cytoscape software, in which bigger degrees were shown in a larger font.

The data discussed in this publication have been deposited in NCBI's Gene Expression Omnibus database (http://www.ncbi.nlm.nih.gov/geo/) under the accession number GSE49249.

## Results

### The pulmonary pathological changes of piglets infected by H1N1 SwIV

**Figure 1:**
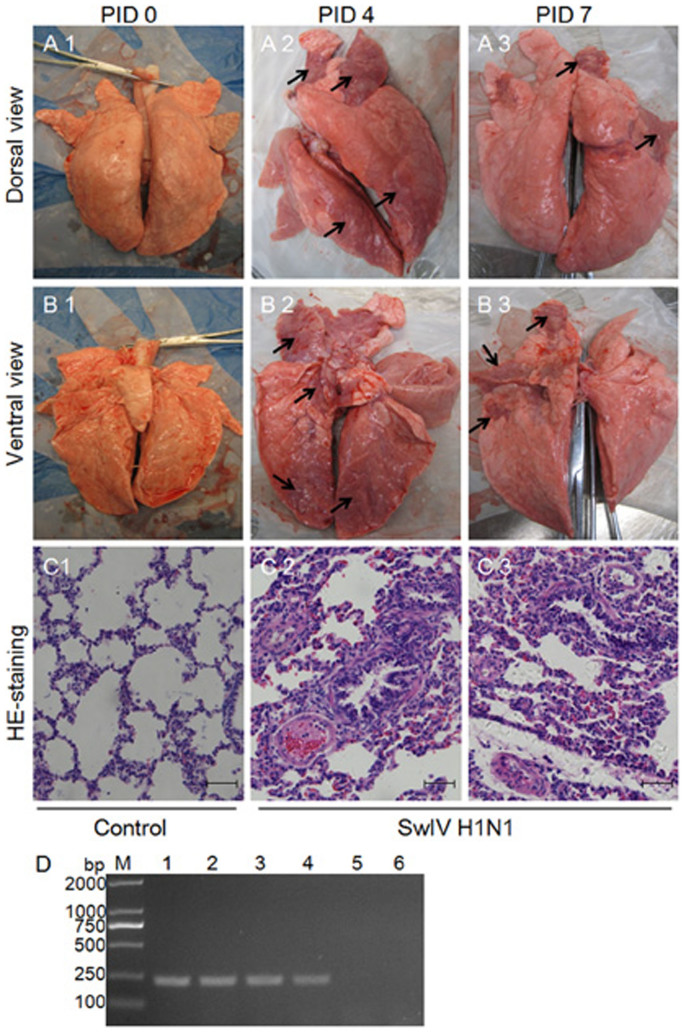
Lung lesions in piglets infected with H1N1 SwIV. (A and B) Piglets were mock infected or infected with H1N1 SwIV. On PID 4 and PID 7, extensive areas of purple-red consolidation indicative of severe pneumonia were observed in lungs of virus-infected piglets, indicated by arrows (A2, B2, A3 and B3). However, on PID 0, similar lesions were not observed in lungs of control piglets (A1 and B1). (C) Microscopic lung lesions in piglets infected with H1N1 SwIV. Lungs of control uninfected piglets show normal alveolar walls, clear air space, and absence of exudation into the alveolar space (C1). Lungs of piglets infected with H1N1 SwIV show exudative interstitial pneumonia on PID 4 and PID 7, illustrating the thickened alveolar walls containing intravascular and extravascular inflammatory cells (C2 and C3). The alveolar spaces are collapsed and contain proteinic debris and an increased population of inflammatory cells. Hematoxylin and eosin staining. Magnification, ×200. Representative lung sections of control and H1N1 SwIV-infected piglets are shown. Similar histological changes were observed in lungs of piglets from another independent experiment. (D) Detection of H1N1 SwIV infection in PAM samples from three groups. Exactly, PB2 gene was amplified by RT-PCR. M, DNA marker; 1 and 2, two samples on PID 4; 3 and 4, two samples on PID 7; 5 and 6, two samples on PID 0.

By monitoring clinical signs of all piglets, it was found that piglets in infected groups showed mild signs, such as coughing, dyspnea and shivering on PID 2-4 and recovered on PID 5-7. The average body temperature of infected piglets started to increase on PID 2, rose to 40.9°C on PID 3, and returned to the initial temperature (about 39°C) until PID 6. As expected, piglets in the control group showed no clinical signs and the body temperature had also no changes. Lung tissues in each group were collected and the macroscopic lesions of lungs tissues were estimated visually (Fig. 1A and B). The pulmonary pathological injury was analyzed using HE-staining. As shown in Fig. 1C, lung tissues from piglets in control group (PID 0) showed a normal structure and no histopathological changes under a light microscope. In infected groups (PID 4 and PID 7), lung tissues indicated widespread alveolar wall thickness caused by alveolar wall capillary congestion, bronchial and alveolar cavity serofluid exudation, mild proliferation of fibroblasts in pulmonary lobule, and bronchial mucosal desquamation. Additionally, the infection of H1N1 SwIV in PAM samples on PID 4 and PID 7 was also determined by RT-PCR. As shown in Fig. 1D, both PAM samples from each group had equivalent amounts of infection.

### Construction of three different small RNA libraries by Solexa sequencing

**Figure 2:**
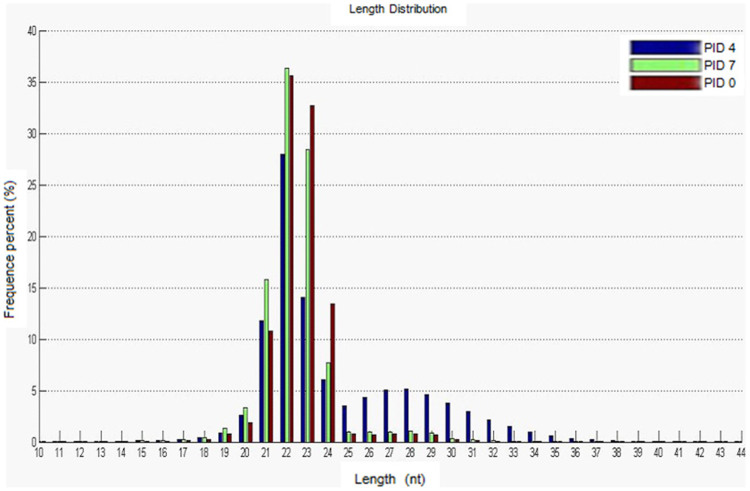
Size and frequency distribution of the sequencing reads. Different colors stand for samples from piglets at different time points post infection with H1N1 SwIV. Nt, nucleotides.

In order to identify DE miRNAs in porcine PAMs at different time points post infection with H1N1 SwIV, three different small RNA libraries were sequenced using Solexa technology. After removing the reads of low quality and masking adaptor sequences, total reads of 18 to 30 nucleotides in length were obtained from three samples. From the size distribution of total reads, we found the length distribution peaked at 21 to 23 nucleotides and more than half of these clean reads (78.97%, 53.78% and 80.51% on PID 0, 4, 7, respectively) were 21 to 23 nucleotides in length, consistent with the common size of miRNA (Fig. 2).

### Category analysis of DE miRNAs

**Figure 3:**
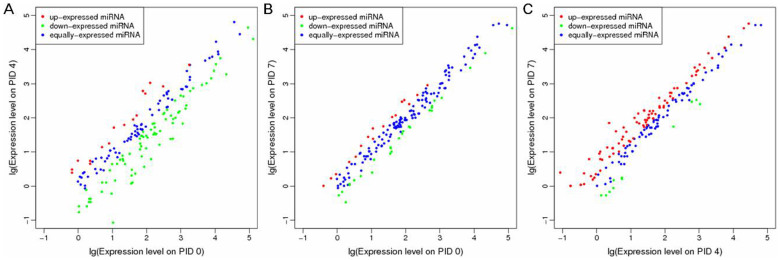
Changing trends of miRNA expression level in samples infected with H1N1 SwIV. (A) Compared with PID 0, changing trends of miRNA expression level on PID 4. (B) Compared with PID 0, changing trends of miRNA expression level on PID 7. (C) Compared with PID 4, changing trends of miRNA expression level on PID 7. In all pictures, DE miRNAs are shown in red and green. The miRNAs without expression changes are shown in blue.

**Figure 4:**
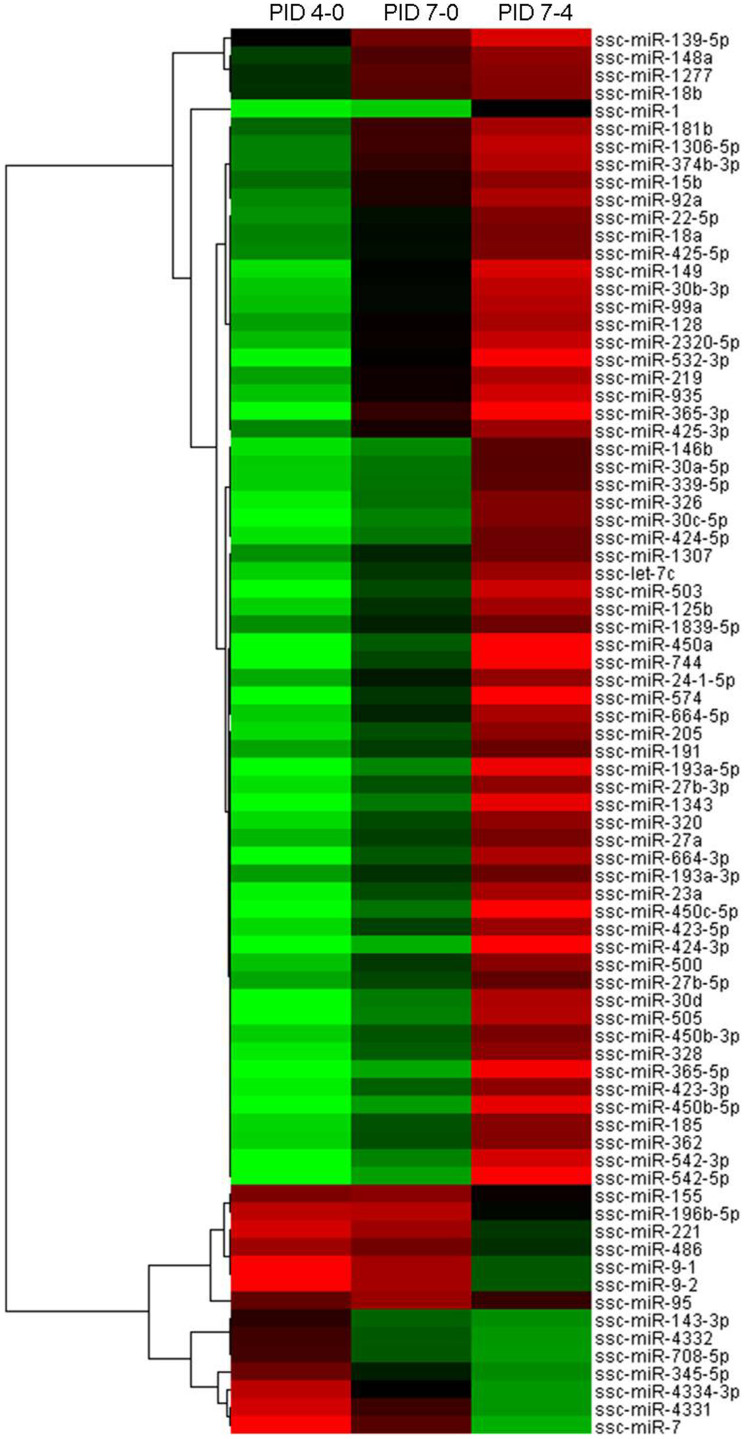
Heat map for clustering of DE miRNAs between samples infected with H1N1 SwIV at different time points. Each row represents one DE miRNA while each column represents each pair-wise comparison. Red color indicates the up-regulated miRNA while green color indicates the down-regulated miRNA and the brighter color indicates the more significant difference. Gray color indicates that miRNA is not expressed at least in one sample.

Based on criteria mentioned above in materials and methods section, known porcine miRNAs in each sample were identified. The numbers of known miRNAs were listed in Table 1. The frequency of individual miRNAs in one sample can be used to compare the relative expression of miRNAs between samples^26^. Thus, we tested the differential expression of miRNAs in samples of PID 4 and PID 7 based on the normalized reads according to the criteria of |fold change| ≥ 1.5 and p < 0.01. The positive value of fold change means the up-regulation of one miRNA while the negative value means the down-regulation of one miRNA. In total, compared with PID 0, 70 known porcine miRNAs were differentially expressed in the sample of PID 4, 8 of which were up-regulated while 62 were down-regulated; 16 known porcine miRNAs were differentially expressed in the sample of PID 7, 6 of which were up-regulated while 10 were down-regulated. 56 known porcine miRNAs were differentially expressed in the sample of PID 7 compared with PID 4, 49 of which were up-regulated while 7 were down-regulated. The changing trends of miRNA expression level were demonstrated by scatter plots (Fig. 3) and details of DE miRNAs were listed in Table S2, S3, and S4. We then conducted the clustering for DE miRNAs in each sample by hierarchical cluster, and the results were shown by heat map (Fig. 4). As shown in the heat map, compared with sample on PID 0, most miRNAs were down-regulated in sample on PID 4, whereas only few miRNAs were differentially expressed in sample on PID 7. Compared with sample on PID 4, a majority of miRNAs were up-regulated in sample on PID 7.

### Validation of DE miRNAs by qRT-PCR

**Figure 5:**
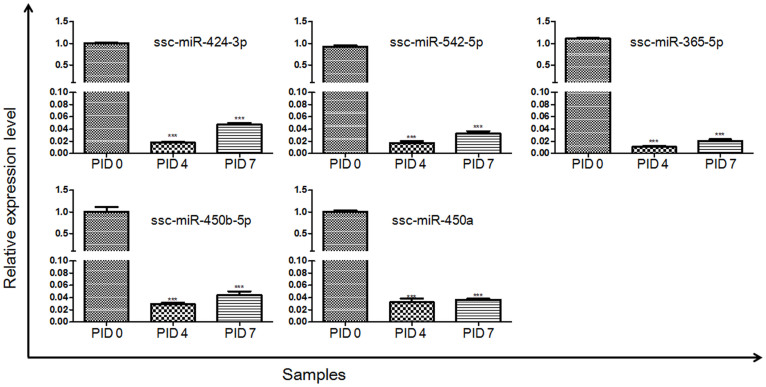
Validation of the sequencing results by qRT-PCR. The relative expression levels of 5 randomly selected miRNAs were detected by qRT-PCR. The X-axis represents the different groups of samples and the Y-axis represents the relative expression levels of miRNAs. ***p < 0.001 versus the control group (PID 0).

To validate the Solexa sequencing results, qRT-PCR was performed separately to investigate the relative expression levels of 5 randomly selected DE miRNAs (ssc-miR-424-3p, ssc-miR-542-5p, ssc-miR-365-5p, ssc-miR-450b-5p, ssc-miR-450a). As shown in Fig. 5, compared with PID 0, the selected miRNAs were down-regulated on PID 4. Compared with PID 4, these miRNAs were up-regulated on PID 7. In general, the changing trends of these miRNAs in the qRT-PCR results were similar to those in the Solexa sequencing results. Therefore, the qRT-PCR results validated the Solexa sequencing results.

To confirm the possibility of using pig as a model to study human influenza, we searched publications to identify DE miRNAs that were conservative between swine and humans after the H1N1 influenza A virus infection. Interestingly, many DE miRNAs, such as miR-542-3p, miR-30d, miR-30c, miR-23a, miR-125b, and so on^27^,^28^,^29^,^30^, were found conservative (Table 2).

### TFs relevant with DE miRNAs

**Figure 6:**
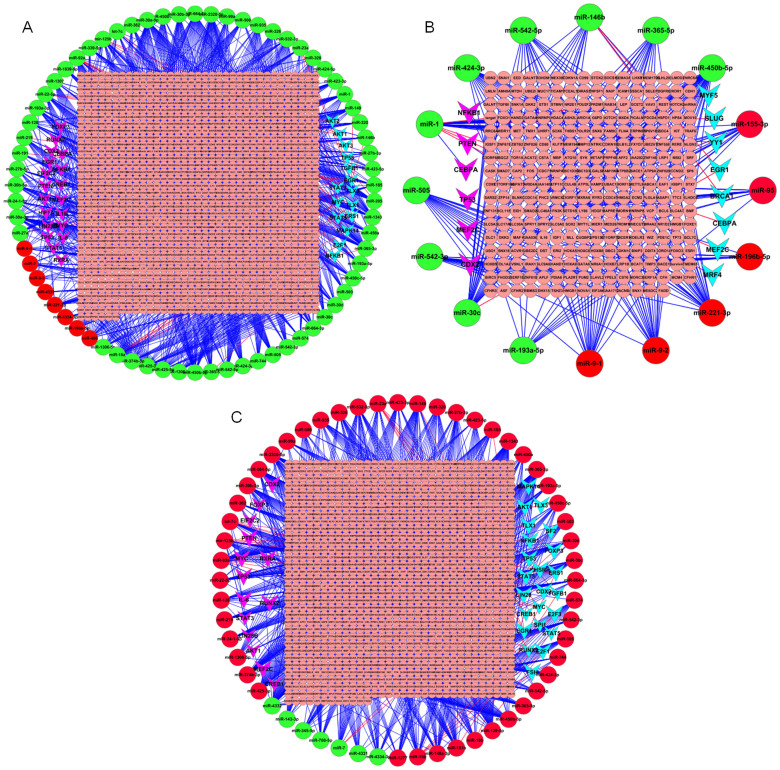
DE miRNA-targets regulatory networks between samples infected with H1N1 SwIV at different time points. (A) DE miRNA-targets regulatory networks between PID 4 and PID 0. (B) DE miRNA-targets regulatory networks between PID 7 and PID 0. (C) DE miRNA-targets regulatory networks between PID 7 and PID 4. In all pictures, larger red circles indicate up-regulated miRNAs while green ones indicate down-regulated miRNAs. Smaller pink circles indicate targets regulated by miRNAs. Pink V symbols indicate target genes coding TFs while blue ones indicate TFs that regulate DE miRNAs. Red lines indicate that TFs regulate miRNAs while blue ones indicate that miRNAs regulate targets.

**Figure 7:**
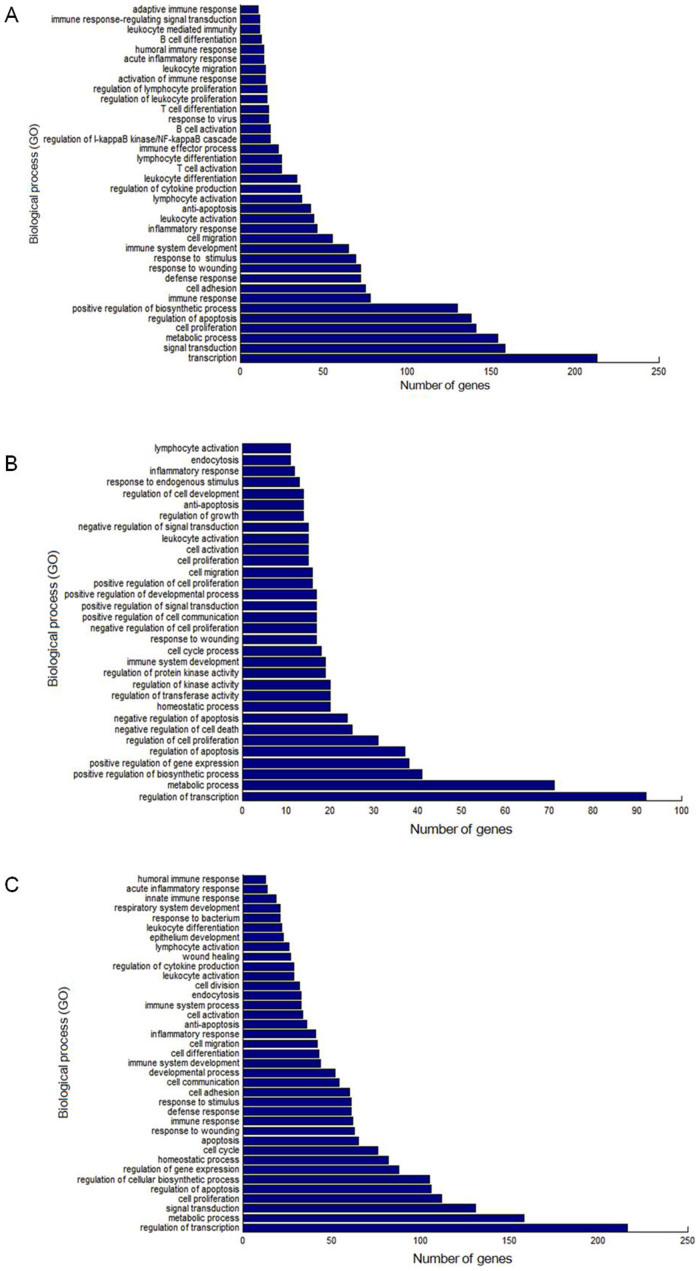
GO annotation on targets of DE miRNAs between samples infected with H1N1 SwIV at different time points. (A) GO annotation on targets of DE miRNAs between PID 4 and PID 0. (B) GO annotation on targets of DE miRNAs between PID 7 and PID 0. (C) GO annotation on targets of DE miRNAs between PID 7 and PID 4. In all pictures, functional classification of targets was performed according to GO biological processes. These targets were sorted by the enrichment of GO categories. The vertical axis is the GO category and the horizontal axis is the enrichment of GO.

The interactions of miRNAs with TFs were downloaded from TransmiR database, in which TFs regulating DE miRNAs were identified. On PID 4, partial DE miRNAs were regulated by TFs, such as AKT1, AKT2, AKT3, E2F1, EGR1, ERS1, STAT3, MAPK14, MYC, TGFB1, TLX1, TLX3, NFKB1, and TP53 (Fig. 6A). On PID 7, partial DE miRNAs were regulated by TFs, such as EGR1, BRCA1, CEBPA, YY1, MYF5, MRF4, SLUG, and MEF2C (Fig. 6B). Compared with PID 4, partial DE miRNAs on PID 7 were regulated by TFs, such as AKT1, E2F1, EGR1, ERS1, MYC, TGFB1, TP53, etc. (Fig. 6C). As shown in Fig. 7, by analysis of GO function annotation, these TFs mainly participate in biological processes including host defense response, inflammatory immune response, apoptosis, metabolism, growth, and transcription, etc.

### MiRNA target prediction and functional annotation analyses

According to the strategy described in materials and methods section, predicted targets of DE miRNAs were obtained. As a result, 1472 targets were predicted for the 70 DE miRNAs between sample on PID 4 and PID 0 (Fig. 6A); 304 targets were predicted for the 16 DE miRNAs between sample on PID 7 and PID 0 (Fig. 6B); 1217 targets were predicted for the 56 DE miRNAs between sample on PID 7 and PID 4 (Fig. 6C). As expected, most miRNAs targeted hundreds of genes, and vice versa. These targets were sorted by the enrichment of GO categories based on the DAVID databases and mainly clustered into different functional groups (Fig. 7). These targets were also analyzed by KEGG pathway annotation. The key pathways, in which DE miRNA targets were involved, were respectively obtained (Table 3, 4, and 5). Among these key pathways, MAPK, focal adhesion, p53, and B cell receptor signaling pathways were closely related to immune and inflammatory responses. Therefore, we analyzed these pathways in detail (Fig. S1, S2, S3, and S4).

### PPI networks regulated by DE miRNAs

**Figure 8:**
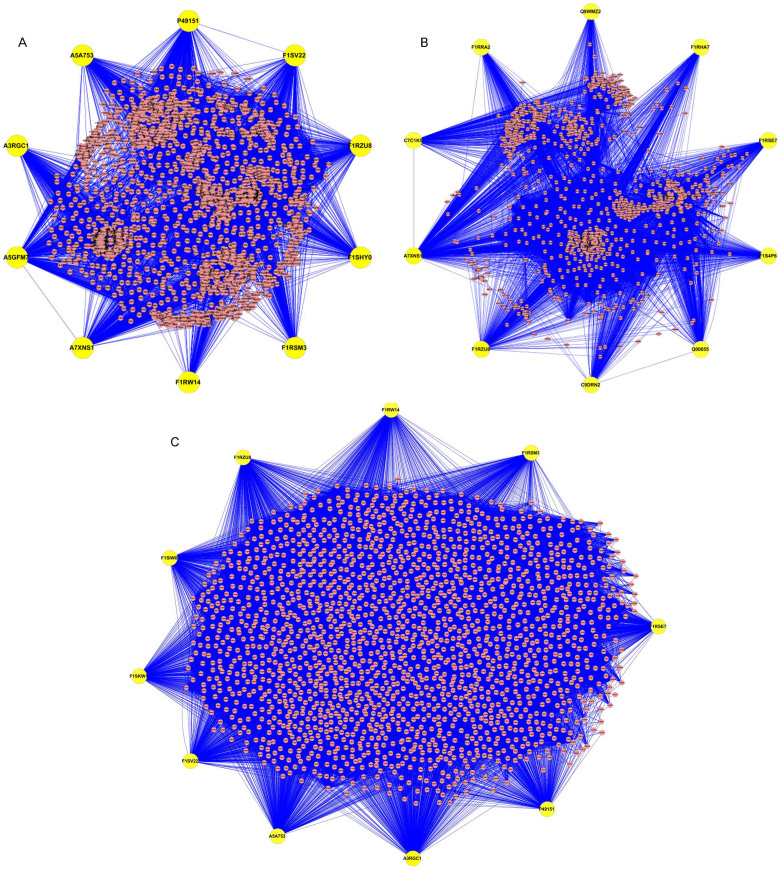
PPI networks mediated by DE miRNAs between samples infected with H1N1 SwIV at different time points. (A) PPI networks mediated by DE miRNAs between PID 4 and PID 0. (B) PPI networks mediated by DE miRNAs between PID 7 and PID 0. (C) PPI networks mediated by DE miRNAs between PID 7 and PID 4. In each network, 10 node proteins with highest degrees were shown. The corresponding gene names to accession numbers in three pictures were described as follows: A7XNS1, NFKB1; A3RGC1, VDR; A5A753, EGFR; P49151, VEGFA; A5GFM7, NCR1; F1RZU8, DST; F1SV22, MACF1; F1SHY0, SOS2; F1RSM3, HELZ; F1RW14, WDFY3; F1RHA7, TGFBI; Q8WMZ2, CD80; Q00655, SYK; C7C1K5, SMAD2; C9DRN2, SOCS1; F1RSE7, MCM4; F1S4P6, EIF3A; F1RRA2, SPRY1; F1SIW0, STAB1; F1SKW1, RC3H2.

The PPI networks regulated by DE miRNAs after infection with H1N1 SwIV at different time points were constructed as described in materials and methods section. Then, the degree distribution analysis was performed, according to which PPI networks containing proteins with the top 10 highest degrees were extracted. As shown in Fig. 8A, in the PPI network mediated by DE miRNAs on PID 4, 10 node proteins were A7XNS1 (Nuclear factor of kappa light polypeptide enhancer in B-cells 1 encoded by gene NFKB1), A3RGC1 (Vitamin D3 receptor encoded by gene VDR), A5A753 (Epidermal growth factor receptor encoded by gene EGFR), P49151 (Vascular endothelial growth factor A encoded by gene VEGFA), A5GFM7 (Natural cytotoxicity triggering receptor 1 encoded by gene NCR1), F1RZU8 (uncharacterized protein encoded by gene DST), F1SV22 (uncharacterized protein encoded by gene MACF1), F1SHY0 (uncharacterized protein encoded by gene SOS2), F1RSM3 (uncharacterized protein encoded by gene HELZ), and F1RW14 (uncharacterized protein encoded by gene WDFY3). Similarly, as shown in Fig. 8B, in the PPI network mediated by DE miRNAs on PID 7, 10 node proteins were A7XNS1, F1RHA7 (Transforming growth factor-beta-induced protein ig-h3 encoded by gene TGFBI), Q8WMZ2 (CD80 encoded by gene CD80), Q00655 (Tyrosine-protein kinase SYK encoded by gene SYK), C7C1K5 (SMAD family member 2 encoded by gene SMAD2), C9DRN2 (Suppressor of cytokine signaling 1 encoded by gene SOCS1), F1RZU8, F1RSE7 (uncharacterized protein encoded by gene MCM4), F1S4P6 (Eukaryotic translation initiation factor 3 subunit A encoded by gene EIF3A), and F1RRA2 (uncharacterized protein encoded by gene SPRY1). As shown in Fig. 8C, in the PPI network mediated by DE miRNAs between PID 4 and PID 7, 10 node proteins were A3RGC1, A5A753, P49151, F1RZU8, F1SV22, F1SIW0 (uncharacterized protein encoded by gene STAB1), F1RSM3, F1RW14, F1RSE7, and F1SKW1 (uncharacterized protein encoded by gene RC3H2).

## Discussion

Recently, the impact of miRNAs expression on understanding molecular mechanisms in gene regulations has been remarkable because a single miRNA has the potential to target hundreds of distinct mRNA molecules and one mRNA molecule can be regulated by multiple miRNAs^31^, which means that miRNAs are attractive candidates as regulators of multiple pathways. However, it has become gradually clear that not all miRNAs are equally significant. Specific miRNAs emerge as principal regulators that control major cell functions in various physiological and pathological settings^32^. Therefore, the search for master miRNAs and related target genes in response to H1N1 SwIV infection and underlying molecular mechanisms is consecutively one of the most important areas in H1N1 SwIV research.

In the present study, we have mainly investigated DE miRNAs in PAMs from piglets at different time points post infection with H1N1 SwIV compared with control piglets. To our knowledge, this is the first study to profile DE miRNAs in H1N1 SwIV infected piglets by Solexa deep sequencing approach. The integration of Solexa deep sequencing technology, DE miRNA analysis, target prediction, functional annotation analyses of targets, and PPI network construction has allowed us to perform a robust comparative genomics and bioinformatics study to reveal the host miRNA molecular signatures associated with H1N1 SwIV infection. Our results also revealed the cellular pathways and PPI networks associated with the differentially expressed host miRNAs during the H1N1 SwIV life cycles. We identified a unique series of cellular miRNAs, providing, for the first time, key molecular insights into unique cellular miRNA-target interactome networks dynamically and temporarily affected by H1N1 SwIV infection.

As shown in Fig. 3A (see also Table S2), compared with PID 0, there are 70 DE miRNAs on PID 4, although most of which are down-regulated, a few are up-regulated; On PID 7, the number of DE miRNAs decreased substantially and there were only 16 DE miRNAs (Fig. 3B; see also Table S3). In addition, compared with PID 4, there are 56 DE miRNAs on PID 7 and a majority of them were up-regulated (Fig. 3C; see also Table S4). Taken together, these results suggest that a large number of miRNAs are down-regulated resulting in the up-regulation of their targets to defend the virus infection during the acute phase (on PID 4). Then, during the recovery phase (on PID 7), expression levels of these down-regulated miRNAs return to normal, leading to the normal expression of immune regulators. However, these results need to be confirmed in the subsequent experiments.

Among these DE miRNAs, consistent with previous studies, many of themselves or their homologs were reported to be closely related with influenza virus infection. For instance, gga-miR-18, gga-miR-193a, gga-miR-193b, gga-miR-30b, miR-1c, gga-miR-146a, gga-miR-24, gga-miR-92, gga-miR-1306, gga-miR-7b, gga-miR-7-1, and gga-miR-7-2 were differentially expressed in chicken lungs infected with avian influenza virus^33^; miR-574-3p, miR-574-5p, miR-744*, miR-30a, miR-30d, miR-205, miR-532-3p, and let-7g were differentially expressed in A549 cells infected with swine-origin H1N1 or avian-origin H7N7 influenza A virus^29^; let-7e, let-7f, miR-193, miR-27a, miR-27b, miR-30a, and miR-30d were differentially expressed in mice infected with recombinant influenza A H1N1 virus strains r1918 or Tx/91^34^; miR-1, miR-23a, miR-30b, miR-30c, miR-30d, and miR-99b were differentially expressed in cynomolgus macaques infected with highly pathogenic H5N1 avian virus^35^; gga-miR-1a-1, gga-miR-1a-2, gga-miR-99a, gga-let-7c, gga-miR-30d, gga-miR-125b, gga-miR-1b, gga-miR-146b, gga-miR-27b, gga-miR-22, gga-miR-30a-3p, gga-miR-30a-5p, gga-miR-30c-1, gga-miR-24, gga-miR-30c-2, gga-miR-1b, and gga-miR-9 were differentially expressed in chicken lung and trachea infected with avian influenza virus^36^.

However, there are still some differences between our results and previous studies. Firstly, some down-regulated miRNAs were reported to be up-regulated in previous studies. For example, gga-miR-18, gga-miR-193a, gga-miR-193b, gga-miR-30b, gga-miR-146a, gga-miR-24, gga-miR-92, gga-miR-7b, gga-miR-7-1, and gga-miR-7-2 are up-regulated after avian influenza virus infection in previous studies whereas in our results these miRNAs are down-regulated on PID 4^33^. In addition, expression patterns of miR-574-3p, miR-574-5p, miR-744*, miR-30a, miR-30d, miR-205 and miR-532-3p are also inconsistent with our results^29^. We speculate that these differences attribute to the different viruses, animals, analysis methods and even the different host regulatory mechanisms response to virus infection. Secondly, we also identified many interesting DE miRNAs that had not been linked to influenza virus infection but other diseases in previous studies. For example, miR-365, miR-503, miR-326, miR-149, miR-185, miR-191, and miR-425 were reported to play important roles in regulating kinds of tumorigenesis such as lung cancer, endometrioid endometrial cancer, brain tumor, colorectal cancer, prostate cancer, breast cancer, and so on^37^,^38^,^39^,^40^,^41^,^42^,^43^. Recently, a study reported that infections with viruses, bacteria, and parasites could contribute to tumorigenesis^44^. Taken together, our results suggest that influenza virus infection may be related to tumorigenesis to some extent. However, this conjecture needs to be explored in the future study.

TFs, an important class of gene regulators, can regulate the expression of miRNAs or be regulated by miRNAs^45^,^46^,^47^. In our study, as shown in Fig. 6, DE miRNAs were regulated by several TFs during H1N1 SwIV infection and vice versa. Furthermore, these TFs were closely related to immune defense (Fig. 7). These results will be helpful to study the roles of the interactions of TFs with miRNAs during H1N1 SwIV infection.

As indicated by previous reports, pig can serve as an excellent animal model to study the pathogenesis of human influenza, which was preliminarily confirmed by our results. As shown in Table 2, many DE miRNAs were identified conservative between pig and humans during the H1N1 influenza A virus infection^27^,^28^,^29^,^30^. This result indicates that in the future study we can use pig as a model to study human influenza, especially these conservative DE miRNAs.

Many studies indicate that inflammatory responses might result in influenza-induced pathogenesis^5^,^10^,^48^. Specially, H1N2 influenza virus was reported to cause pathological damage to lungs due to its induction of pro-inflammatory cytokines such as IL-1, IL-8, and TNF-α^10^. Interestingly, on both PID 4 and PID 7, there are several up-regulated DE miRNAs whose targets are genes encoding immune and inflammatory cytokines (Table S5, S6, S7, and S8), base on which we can speculate that these up-regulated DE miRNAs serve as negative regulators for immune and inflammatory cytokines during H1N1 SwIV infection to avoid too severe porcine lung damage.

As shown in Table 3, 4, and 5, by KEGG pathway annotation, DE miRNAs at different time points were found to be involved in many key pathways such as MAPK signaling pathway, focal adhesion, cytokine-cytokine receptor interaction, Jak-STAT signaling pathway, chemokine signaling pathway, T cell receptor signaling pathway, and so on, most of which were reported to be related with the influenza virus infection^49^.

Take MAPK signaling pathway for instance, as shown in Fig. S1A, on PID 4, there are 38 DE miRNAs involved in MAPK signaling pathway and most DE miRNAs such as miR-450b-5p, miR-146b, miR-1343, miR-128, and miR-30a-5p were down-regulated while their targets such as MEF2C, NFKB1, TGFBR1, EGFR, JUN, and MAPK1 are key factors in MAPK signaling pathway (see map 04010 in KEGG database). As a result, on PID 4, MAPK signaling pathway was activated. As shown in Fig. S1B, the number of DE miRNAs involved in MAPK signaling pathway significantly decreased on PID 7, which suggests that abnormal cell proliferation, differentiation and apoptosis were reduced. Compared with PID 4, most of DE miRNAs involved in MAPK signaling pathway were up-regulated on PID 7 (Fig. S1C), which means MAPK signaling pathway was inhibited. Other signaling pathways also appear similar situations. Taken together, it is therefore intriguing to speculate that during the acute phase of H1N1 SwIV infection, the host turns on many signaling pathways mentioned above to defend the virus infection; however, during the recovery phase, most of signaling pathways are turned off to avoid too severe tissue damage.

Biological processes inside cells are governed by the well-organized protein-protein interaction networks, which act as molecular machines performing different functionality. One key aim of post-genomic biology is to reconstruct the complete molecular interaction networks within cells and on the basis of which to understand the principles on the construction, function and evolution of life. In this study, we constructed the PPI networks regulated by DE miRNAs after infection with H1N1 SwIV at different time points. According to degree distribution analysis, three sub-networks containing proteins with the top 10 highest degrees were extracted. As shown in Fig. 8, most of these node proteins are related to virus infection such as Nuclear factor of kappa light polypeptide enhancer in B-cells 1, Natural cytotoxicity triggering receptor 1, Suppressor of cytokine signaling 1, Epidermal growth factor receptor, Vitamin D3 receptor, CD80, Tyrosine-protein kinase SYK, and SMAD family member 2^50^,^51^,^52^,^53^,^54^,^55^,^56^,^57^,^58^. Therefore, as the node proteins in PPI networks regulated by DE miRNAs, these proteins may be closely related with H1N1 SwIV infection. Furthermore, the PPI networks may provide useful information to study the interaction between host and H1N1 SwIV virus.

In this study we presented the profile of DE miRNAs in porcine PAMs during the H1N1 SwIV virus infection for the first time to our knowledge. Our results suggest that most host miRNAs are down-regulated to defend the infection of H1N1 SwIV during the acute phase of swine influenza whereas their expression levels gradually return to normal during the recovery phase to avoid the occurrence of too severe porcine lung damage. Furthermore, we also obtained targets of DE miRNAs, performed GO and KEGG analyses for these targets and constructed PPI networks regulated by DE miRNAs. The identification and functional annotation analyses of these DE miRNAs will be very useful for further investigating the functions and regulatory mechanisms of miRNAs in piglets infected with influenza virus.

## Supplementary Material

Supplementary Information supplementary tables and figures

## Figures and Tables

**Table 1:** Numbers of known miRNA after infection with H1N1 SwIV at different time points

Different time points	Mature miRNAs	miRNA precursors	Reads matched to miRNA precursors
PID 0	214	195	11710898
PID 4	204	184	5634260
PID 7	207	187	10911797

**Table 2:** Differentially expressed miRNAs that are conservative between swine and humans when infected by H1N1 influenza A virus

reference miRNAs	This study	Ref. 27	Ref. 28	Ref. 29	Ref. 30
miR-542-3p	+	+	-	-	-
miR-744	+	+	-	-	-
miR-30d	+	+	-	+	-
miR-30c	+	+	-	-	-
miR-532-3p	+	-	-	+	-
miR-23a	+	+	-	-	+
miR-423-3p	+	+	-	-	-
miR-328	+	-	-	+	-
miR-205	+	-	-	+	-
miR-423-5p	+	+	-	-	-
miR-185	+	+	-	-	-
let-7c	+	+	-	-	-
miR-125b	+	+	+	-	-
miR-339-5p	+	+	-	-	-
miR-99a	+	+	-	-	-
miR-191	+	+	-	-	-
miR-92a	+	+	-	-	-
miR-18a	+	+	-	-	-
miR-95	+	-	+	-	-
miR-30c	+	+	-	-	-
miR-500	+	-	-	-	+
miR-7	+	-	-	-	+

+/-: Differentially expressed miRNAs that are conservative or not between swine and humans when infected by H1N1 influenza A virus.

**Table 3:** Summary of key pathways in which DE miRNA targets were involved after infection with H1N1 SwIV on PID 4

KEGG pathway (PID 4-0)	p-value	Gene number	Related miRNA number
MAPK signaling pathway	1.2E-9	55	38
Focal adhesion	1.9E-12	52	33
Cytokine-cytokine receptor interaction	1.0E-5	45	34
Jak-STAT signaling pathway	6.0E-9	39	32
Cell cycle	1.5E-7	32	26
Chemokine signaling pathway	6.2E-4	32	24
T cell receptor signaling pathway	9.9E-7	29	19
Cell adhesion molecules (CAMs)	4.8E-5	27	26
ErbB signaling pathway	1.7E-7	26	19
Wnt signaling pathway	2.4E-3	26	20
P53 signaling pathway	4.3E-9	25	24
Toll-like receptor signaling pathway	3.7E-5	25	19
Natural killer cell mediated cytotoxicity	4.8E-3	23	18
B cell receptor signaling pathway	1.3E-5	22	18
Apoptosis	3.7E-4	19	18
TGF-beta signaling pathway	1.1E-3	19	18
Epithelial cell signaling in Helicobacter pylori infection	4.9E-3	14	13
RIG-I-like receptor signaling pathway	4.1E-2	12	13
mTOR signaling pathway	3.3E-2	10	12

**Table 4:** Summary of key pathways in which DE miRNA targets were involved after infection with H1N1 SwIV on PID 7

KEGG pathway (PID 7-0)	p-value	Gene number	Related miRNA number
MAPK signaling pathway	3.8E-2	10	8
Focal adhesion	2.1E-2	9	6
P53 signaling pathway	1.7E-5	9	5
Toll-like receptor signaling pathway	7.3E-3	7	6
Cell cycle	2.0E-2	7	6
Cell adhesion molecules (CAMs)	2.5E-2	7	7
B cell receptor signaling pathway	3.9E-2	5	7

**Table 5:** Summary of key pathways in which DE miRNA targets were involved after infection with H1N1 SwIV on PID 7 comparing with PID 4

KEGG pathway (PID 7-4)	p-value	Gene number	Related miRNA number
MAPK signaling pathway	9.1E-7	42	30
Focal adhesion	8.8E-7	35	25
Jak-STAT signaling pathway	5.7E-8	32	23
Cytokine-cytokine receptor interaction	8.6E-3	30	23
Cell cycle	1.4E-5	24	22
Chemokine signaling pathway	1.1E-2	23	18
ErbB signaling pathway	1.4E-6	21	18
T cell receptor signaling pathway	3.8E-6	24	15
Cell adhesion molecules (CAMs)	7.5E-4	21	16
P53 signaling pathway	5.1E-5	16	17
B cell receptor signaling pathway	5.8E-4	14	13
Apoptosis	7.0E-3	14	14
Toll-like receptor signaling pathway	2.3E-2	14	14
mTOR signaling pathway	2.7E-2	9	10
