# Supplementary material for: Integrative analysis of differentially expressed microRNAs of pulmonary alveolar macrophages from piglets during H1N1 swine influenza A virus infection
Source: Sci Rep. 2015 Feb 2;5:8167. doi: 10.1038/srep08167 (PMC5389138; doi:10.1038/srep08167)
Supplement: Supplementary Information — supplementary tables and figures [file srep08167-s1.doc]

Integrative analysis of differentially expressed microRNAs of pulmonary alveolar macrophages from piglets during H1N1 swine influenza A virus infection

Pengfei Jiang^a, b§***^, Na Zhou^a§^, Xinyu Chen^a§^, Xing Zhao^a^, Dengyun Li^a^, Fen Wang^a^, Lijun Bi^c**^, Deli Zhang^a*^

Table S1. Quantitative real-time PCR primer sequences

| MiRNAs | Primer sequences (5′→3′) | |
| --- | --- | --- |
| ssc-miR-424-3p  ssc-miR-542-5p  ssc-miR-365-5p  ssc-miR-450b-5p  ssc-miR-450a  U6 forward  U6 reverse | CAAAACGTGAGGCGCTGCTAT  TCGGGGATCATCATGTCACGA  GAGGGACTTTCAGGGGCAGCTGT  TTTTGCAATATGTTCCTGAATA  TTTTGCGATGTGTTCCTAATAT  CTCGCTTCGGCAGCACA  AACGCTTCACGAATTTGCGT |  |

Table S2. Differentially expressed miRNAs after infection with H1N1 SwIV on PID 4 compared with PID 0.

| miRNA name | Normalized miRNA expression level | | Fold change (log2 PID 4/PID 0) | | | p-value |
| --- | --- | --- | --- | --- | --- | --- |
|  | PID 4 | PID 0 |  | | |  |
| ssc-miR-424-3p | 0.0853 | 10.3986 | -6.92962785 | 4.72E-38 | | |
| ssc-miR-542-5p | 1.2801 | 38.3695 | -4.90563154 | 2.4E-121 | | |
| ssc-miR-365-5p | 1.1948 | 35.5395 | -4.89458234 | 2.3E-112 | | |
| ssc-miR-450b-5p | 24.6642 | 579.7539 | -4.55495022 | 0 | | |
| ssc-miR-450a | 3.6698 | 84.7024 | -4.5286295 | 2E-254 | | |
| ssc-miR-450c-5p | 6.1447 | 134.4576 | -4.45166492 | 0 | | |
| ssc-miR-193a-5p | 24.1521 | 499.0661 | -4.36901037 | 0 | | |
| ssc-miR-365-3p | 2.3043 | 46.1355 | -4.32347683 | 2.2E-135 | | |
| ssc-miR-1343 | 2.0482 | 36.3292 | -4.14870109 | 4.3E-104 | | |
| ssc-miR-542-3p | 67.6771 | 1135.683 | -4.06874876 | 0 | | |
| ssc-miR-574 | 0.5974 | 9.4772 | -3.98769174 | 2.06E-27 | | |
| ssc-miR-744 | 16.3859 | 235.8767 | -3.8475061 | 0 | | |
| ssc-miR-505 | 2.987 | 37.119 | -3.6353887 | 1.64E-96 | | |
| ssc-miR-30d | 1885.914 | 21245.88 | -3.49384775 | 0 | | |
| ssc-miR-503 | 7.4249 | 71.2764 | -3.26298097 | 1.1E-167 | | |
| ssc-miR-664-3p | 1.4508 | 12.0439 | -3.05338208 | 6.89E-28 | | |
| ssc-miR-30c | 70.1521 | 578.4376 | -3.04360314 | 0 | | |
| ssc-miR-532-3p | 12.8868 | 96.6147 | -2.90634865 | 5.1E-203 | | |
| ssc-miR-23a | 187.4136 | 1373.6 | -2.8736645 | 0 | | |
| ssc-miR-326 | 0.6827 | 4.8702 | -2.83465736 | 2.82E-11 | | |
| ssc-miR-423-3p | 99.8515 | 699.0085 | -2.80745399 | 0 | | |
| ssc-miR-1 | 0.256 | 1.777 | -2.79522797 | 9.52E-05 | | |
| ssc-miR-328 | 0.3414 | 2.3035 | -2.75429263 | 8.77E-06 | | |
| ssc-miR-424-5p | 92.6827 | 590.5473 | -2.67168064 | 0 | | |
| ssc-miR-146b | 20350.94 | 129226.9 | -2.66673897 | 0 | | |
| ssc-miR-27b-3p | 232.5602 | 1446.983 | -2.63737135 | 0 | | |
| ssc-miR-149 | 0.1707 | 1.053 | -2.62497047 | 0.004504 | | |
| ssc-miR-320 | 41.3061 | 247.4599 | -2.582768 | 0 | | |
| ssc-miR-205 | 0.3414 | 2.0402 | -2.57917562 | 5.66E-05 | | |
| Table S2. Cont. | | | | | | |
| miRNA name | Normalized miRNA expression level | | Fold change (log2 PID 4/PID 0) | | p-value | |
|  | PID 4 | PID 0 |  |  | | |
| ssc-miR-423-5p | 153.7031 | 915.2727 | -2.57405539 | 0 | | |
| ssc-miR-362 | 34.1373 | 191.9131 | -2.49103233 | 0 | | |
| ssc-let-7c | 1032.823 | 5663.542 | -2.45511124 | 0 | | |
| ssc-miR-125b | 33.3692 | 182.6333 | -2.45236084 | 2.7E-318 | | |
| ssc-miR-339-5p | 11.8627 | 64.1027 | -2.43395271 | 5.9E-112 | | |
| ssc-miR-450b-3p | 0.4267 | 2.3035 | -2.43253358 | 3.26E-05 | | |
| ssc-miR-30a-5p | 1436.923 | 7685.013 | -2.41906475 | 0 | | |
| ssc-miR-664-5p | 4.4378 | 23.4955 | -2.40446791 | 1.69E-41 | | |
| ssc-miR-30b-3p | 1.2801 | 6.5814 | -2.36213799 | 2.19E-12 | | |
| ssc-miR-935 | 0.4267 | 2.106 | -2.30321142 | 0.000123 | | |
| ssc-miR-500 | 32.5158 | 159.3352 | -2.29285219 | 5.8E-257 | | |
| ssc-miR-99a | 35.7588 | 168.8782 | -2.23961288 | 1.1E-264 | | |
| ssc-miR-2320-5p | 14.8497 | 69.0387 | -2.21697151 | 5.7E-108 | | |
| ssc-miR-27a | 924.2667 | 4118.628 | -2.15578282 | 0 | | |
| ssc-miR-30a-3p | 116.2374 | 513.0187 | -2.14193708 | 0 | | |
| ssc-miR-24-1-5p | 26.2857 | 106.4209 | -2.01743145 | 3.6E-147 | | |
| ssc-miR-30b-5p | 91.0612 | 365.0034 | -2.00300153 | 0 | | |
| ssc-miR-27b-5p | 1.2801 | 5.0018 | -1.96619086 | 5.08E-08 | | |
| ssc-miR-191 | 2396.693 | 9261.32 | -1.95017283 | 0 | | |
| ssc-miR-219 | 5.5473 | 20.7314 | -1.90195989 | 6.89E-28 | | |
| ssc-miR-128 | 17.4954 | 64.8924 | -1.89107387 | 3.04E-83 | | |
| ssc-miR-193a-3p | 8.7903 | 31.5906 | -1.84551103 | 4.18E-40 | | |
| ssc-miR-22-5p | 29.8701 | 97.6019 | -1.70820717 | 1.9E-108 | | |
| ssc-miR-1307 | 52.9128 | 172.6296 | -1.70599119 | 4.9E-190 | | |
| ssc-miR-1839-5p | 6.2301 | 19.8758 | -1.6736857 | 9.21E-23 | | |
| ssc-miR-92a | 739.2427 | 2269.524 | -1.61826943 | 0 | | |
| ssc-miR-425-5p | 615.0683 | 1874.443 | -1.60764358 | 0 | | |
| ssc-miR-1306-3p | 4.5232 | 13.6893 | -1.59763298 | 3.39E-15 | | |
| ssc-miR-425-3p | 44.7198 | 133.3388 | -1.57611101 | 8.3E-132 | | |
| ssc-miR-18a | 27.5658 | 80.3587 | -1.54357475 | 7.74E-78 | | |
| Table S2. Cont. |  |  |  |  | | |
| miRNA name | Normalized miRNA expression level | | Fold change (log2 PID 4/PID 0) | | p-value | |
|  | PID 4 | PID 0 |  |  | | |
| ssc-miR-374b-3p | 2.9017 | 8.4242 | -1.53764132 | 2.04E-09 | | |
| ssc-miR-1306-5p | 5.8887 | 16.7825 | -1.51093656 | 5.23E-17 | | |
| ssc-miR-486 | 2.475 | 0.6581 | 1.9110498 | 0.000108 | | |
| ssc-miR-4334-3p | 3.0724 | 0.6581 | 2.22298733 | 1.85E-06 | | |
| ssc-miR-196b-5p | 51.8033 | 11.0567 | 2.22812314 | 5.62E-87 | | |
| ssc-miR-4331 | 5.5473 | 0.9872 | 2.49037145 | 5.44E-12 | | |
| ssc-miR-221 | 516.2409 | 91.0863 | 2.50273846 | 0 | | |
| ssc-miR-9-1 | 612.5081 | 77.4629 | 2.98315151 | 0 | | |
| ssc-miR-9-2 | 612.5081 | 77.4629 | 2.98315151 | 0 | | |
| ssc-miR-7 | 1056.634 | 126.1651 | 3.06609095 | 0 | | |

The positive value of fold change means up-regulation of miRNA.

The negative value means down-regulation of miRNA.

Table S3. Differentially expressed miRNAs after infection with H1N1 SwIV on PID 7 compared with PID 0.

| miRNA name | Normalized miRNA expression level | | Fold change (log2 PID 7/PID 0) | p-value |
| --- | --- | --- | --- | --- |
|  | PID 7 | PID 0 |  |  |
| ssc-miR-196b-5p | 11.0567 | 48.7561 | 2.14066187 | 9.39E-86 |
| ssc-miR-9-1 | 77.4629 | 298.2169 | 1.9447846 | 0 |
| ssc-miR-9-2 | 77.4629 | 298.2169 | 1.9447846 | 0 |
| ssc-miR-221-3p | 91.0863 | 332.8398 | 1.86952197 | 0 |
| ssc-miR-95 | 7.9635 | 27.6578 | 1.79621186 | 1.82E-39 |
| ssc-miR-155-3p | 4.936 | 15.1475 | 1.61766541 | 1.8E-19 |
| ssc-miR-505 | 37.119 | 12.9836 | -1.51546739 | 2.95E-41 |
| ssc-miR-30c | 578.4376 | 199.3522 | -1.53684181 | 0 |
| ssc-miR-542-3p | 1135.683 | 383.6246 | -1.56579318 | 0 |
| ssc-miR-193a-5p | 499.0661 | 166.758 | -1.58147493 | 0 |
| ssc-miR-146b | 129226.9 | 41919.49 | -1.62421302 | 0 |
| ssc-miR-450b-5p | 579.7539 | 162.5654 | -1.83442039 | 0 |
| ssc-miR-542-5p | 38.3695 | 10.2787 | -1.90030215 | 5.6E-58 |
| ssc-miR-365-5p | 35.5395 | 8.9262 | -1.99330535 | 3.02E-57 |
| ssc-miR-424-3p | 10.3986 | 2.502 | -2.05523561 | 1.67E-18 |
| ssc-miR-1 | 1.777 | 0.3381 | -2.39392176 | 9.06E-05 |

The positive value of fold change means up-regulation of miRNA.

The negative value means down-regulation of miRNA.

Table S4. Differentially expressed miRNAs after infection with H1N1 SwIV on PID 7 compared with PID 4.

| miRNA name | Normalized miRNA expression level | | Fold change (log2 PID 7/PID 4) | p-value |
| --- | --- | --- | --- | --- |
|  | PID 7 | PID 4 |  |  |
| ssc-miR-365-3p | 2.3043 | 70.5983 | 4.93723298 | 3.5E-221 |
| ssc-miR-424-3p | 0.0853 | 2.502 | 4.87439224 | 8.34E-09 |
| ssc-miR-450a | 3.6698 | 40.3032 | 3.45712105 | 1.2E-99 |
| ssc-miR-574 | 0.5974 | 6.1537 | 3.36468497 | 5.25E-16 |
| ssc-miR-450c-5p | 6.1447 | 52.2049 | 3.08677074 | 3.4E-116 |
| ssc-miR-744 | 16.3859 | 133.6228 | 3.02763937 | 3.1E-289 |
| ssc-miR-542-5p | 1.2801 | 10.2787 | 3.00532939 | 1.77E-23 |
| ssc-miR-532-3p | 12.8868 | 99.7437 | 2.95233166 | 4.3E-211 |
| ssc-miR-365-5p | 1.1948 | 8.9262 | 2.90127699 | 6.57E-20 |
| ssc-miR-193a-5p | 24.1521 | 166.758 | 2.78753543 | 0 |
| ssc-miR-1343 | 2.0482 | 13.5922 | 2.73035048 | 6.71E-28 |
| ssc-miR-450b-5p | 24.6642 | 162.5654 | 2.72052983 | 3.4E-315 |
| ssc-miR-139-5p | 0.1707 | 1.0143 | 2.57094946 | 0.006109 |
| ssc-miR-149 | 0.1707 | 1.0143 | 2.57094946 | 0.006109 |
| ssc-miR-542-3p | 67.6771 | 383.6246 | 2.50295558 | 0 |
| ssc-miR-935 | 0.4267 | 2.3668 | 2.47164378 | 2.28E-05 |
| ssc-miR-503 | 7.4249 | 39.086 | 2.39620845 | 3.82E-67 |
| ssc-miR-2320-5p | 14.8497 | 74.4528 | 2.32589232 | 4.7E-122 |
| ssc-miR-30b-3p | 1.2801 | 6.1537 | 2.2651976 | 4.39E-11 |
| ssc-miR-1306-5p | 5.8887 | 27.9282 | 2.24570151 | 5.83E-45 |
| ssc-miR-374b-3p | 2.9017 | 12.8483 | 2.14660721 | 2.07E-20 |
| ssc-miR-99a | 35.7588 | 157.1556 | 2.13582345 | 1.9E-230 |
| ssc-miR-505 | 2.987 | 12.9836 | 2.11992132 | 2.58E-20 |
| ssc-miR-219 | 5.5473 | 23.3299 | 2.07232247 | 2.24E-34 |
| ssc-miR-30d | 1885.914 | 7795.295 | 2.04733991 | 0 |
| ssc-miR-664-3p | 1.4508 | 5.8832 | 2.01975243 | 2.02E-09 |
| ssc-miR-92a | 739.2427 | 2981.493 | 2.01191476 | 0 |
| ssc-miR-664-5p | 4.4378 | 17.7848 | 2.00272819 | 1.7E-25 |
| ssc-miR-23a | 187.4136 | 737.6303 | 1.97667227 | 0 |
| Table S4. Cont. |  |  |  |  |
| miRNA name | Normalized miRNA expression level | | Fold change (log2 PID 7/PID 4) | p-value |
|  | PID 7 | PID 4 |  |  |
| ssc-miR-128 | 17.4954 | 68.2315 | 1.96346228 | 5.85E-91 |
| ssc-miR-181b | 140.3042 | 545.4461 | 1.95887844 | 0 |
| ssc-miR-125b | 33.3692 | 124.1556 | 1.89556033 | 1.2E-156 |
| ssc-miR-425-3p | 44.7198 | 159.7929 | 1.83721767 | 7E-193 |
| ssc-let-7c | 1032.823 | 3639.058 | 1.81697153 | 0 |
| ssc-miR-423-5p | 153.7031 | 540.2392 | 1.81345206 | 0 |
| ssc-miR-24-1-5p | 26.2857 | 87.1659 | 1.7294857 | 8.6E-98 |
| ssc-miR-320 | 41.3061 | 133.9609 | 1.69738522 | 1.7E-145 |
| ssc-miR-148a-3p | 7345.744 | 23702.77 | 1.69007482 | 0 |
| ssc-miR-27b-3p | 232.5602 | 745.0012 | 1.67963853 | 0 |
| ssc-miR-15b | 68.7013 | 220.0448 | 1.67938798 | 3E-234 |
| ssc-miR-423-3p | 99.8515 | 319.4505 | 1.67773639 | 0 |
| ssc-miR-500 | 32.5158 | 99.1351 | 1.60825503 | 2.4E-100 |
| ssc-miR-1277 | 2.3896 | 7.2356 | 1.59834351 | 1.34E-08 |
| ssc-miR-185 | 142.6938 | 427.3766 | 1.58258527 | 0 |
| ssc-miR-362 | 34.1373 | 100.8933 | 1.56340951 | 4.13E-98 |
| ssc-miR-18b | 3.0724 | 8.9262 | 1.53868008 | 7.88E-10 |
| ssc-miR-326 | 0.6827 | 1.9611 | 1.52233945 | 0.00513 |
| ssc-miR-22-5p | 29.8701 | 85.7458 | 1.52136394 | 1.77E-80 |
| ssc-miR-30c | 70.1521 | 199.3522 | 1.50676133 | 4.5E-182 |
| ssc-miR-345-5p | 5.462 | 1.7582 | -1.63533012 | 2.73E-07 |
| ssc-miR-143-3p | 177.5138 | 55.3155 | -1.68217548 | 5.8E-198 |
| ssc-miR-4331 | 5.5473 | 1.6229 | -1.77321164 | 4.04E-08 |
| ssc-miR-4332 | 1.8776 | 0.541 | -1.79518925 | 0.001389 |
| ssc-miR-4334-3p | 3.0724 | 0.8791 | -1.80526686 | 3.73E-05 |
| ssc-miR-708-5p | 2.3896 | 0.6762 | -1.82124722 | 0.000261 |
| ssc-miR-7 | 1056.634 | 254.0591 | -2.05623969 | 0 |

The positive value of fold change means up-regulation of miRNA.

The negative value means down-regulation of miRNA.

Table S5. Summary of differentially expressed miRNAs whose targets are associated with immune responses on PID 4.

| miRNA name | Target genes | Fold change |
| --- | --- | --- |
| ssc-miR-1 | THBS1, HSPD1 | -2.79523 |
| ssc-miR-1306-3p | IL13 | -1.59763 |
| ssc-miR-1307 | LCP2, IL32, BCL3, CD4, CD79B | -1.70599 |
| ssc-miR-1343 | IL10 | -4.1487 |
| ssc-miR-146b | TRAF6 | -2.66674 |
| ssc-miR-1839-5p | IL13, MIF | -1.67369 |
| ssc-miR-18a | THBS1, TNFSF11 | -1.54357 |
| ssc-miR-191 | IL1A | -1.95017 |
| ssc-miR-205 | ZEB1, LYN, INPPL1, VEGFA, IL32 | -2.57918 |
| ssc-miR-219 | FASLG, CD4 | -1.90196 |
| ssc-miR-221-3p | BNIP3, TICAM1, ICAM1, TP53, CORO1A, TNFSF10, ETS1 | 2.502738 |
| ssc-miR-2320-5p | CD8A, IL13, CD97, LTF, CD83, SEMA4D, CCL1, IL23R, CD180 | -2.21697 |
| ssc-miR-23a | CXCL12, IL6R | -2.87366 |
| ssc-miR-27a | PPARG, NOTCH1 | -2.15578 |
| ssc-miR-27b-3p | PPARG, CXCL12, IL6R, NOTCH1 | -2.63737 |
| ssc-miR-27b-5p | PRKRA | -1.96619 |
| ssc-miR-30a-3p | IL1B, CCR7, IL18R1 | -2.14194 |
| ssc-miR-30a-5p | IGF1R, THBS1 | -2.41906 |
| ssc-miR-30b-3p | APOBEC3F, PVRL1, CCBP2, THBS1, CCL16 | -2.36214 |
| ssc-miR-30b-5p | GALNT2, BNIP3L | -2.003 |
| ssc-miR-30d | TP53 | -3.49385 |
| ssc-miR-326 | MSH3, NOTCH1 | -2.83466 |
| ssc-miR-328 | CFH | -2.75429 |
| ssc-miR-362 | DDX58 | -2.49103 |
| ssc-miR-365-3p | BCL2 | -4.32348 |
| ssc-miR-365-5p | BLNK | -4.89458 |
| ssc-miR-374b-3p | VEGFA | -1.53764 |
| ssc-miR-425-3p | MLH1, CD300LG | -1.57611 |
| ssc-miR-4331 | FAS, SERPING1, CYP27B1, DMBT1 | 2.490371 |
| ssc-miR-4334-3p | TNFSF13, CTNNBL1, CCR7, IL7R, PSMB8 | 2.222987 |
| ssc-miR-450b-3p | TLR9 | -2.43253 |
| Table S5. Cont. |  |  |
| miRNA name | Target genes | Fold change |
| ssc-miR-450b-5p | IL16 | -4.55495 |
| ssc-miR-505 | CFHR1, CFH | -3.63539 |
| ssc-miR-532-3p | CSF3 | -2.90635 |
| ssc-miR-542-5p | LY86, MBP | -4.90563 |
| ssc-miR-574 | IL6 | -3.98769 |
| ssc-miR-664-3p | DDX58 | -3.05338 |
| ssc-miR-664-5p | TREM2 | -2.40447 |
| ssc-miR-7 | RELA, IGF1R | 3.066091 |
| ssc-miR-9-1 | POU2F2, ETS1 | 2.983152 |
| ssc-miR-9-2 | POU2F2, ETS1 | 2.983152 |
| ssc-miR-92a | RAG1 | -1.61827 |
| ssc-miR-935 | EOMES | -2.30321 |
| ssc-miR-99a | IGF1R | -2.23961 |
| ssc-mir-125b | TP53, CLU, LIF, CEBPG | -2.45236 |

Table S6. Summary of differentially expressed miRNAs whose targets are associated with inflammatory responses on PID 4.

| miRNA name | Target genes | Fold change |
| --- | --- | --- |
| ssc-miR-1 | THBS1, HDAC4 | -2.79523 |
| ssc-miR-1306-3p | IL13 | -1.59763 |
| ssc-miR-1343 | IL10, CD40 | -4.1487 |
| ssc-miR-146b | NFKB1 | -2.66674 |
| ssc-miR-1839-5p | IL13, MIF, SMAD1 | -1.67369 |
| ssc-miR-185 | SGMS1, AKT1 | -2.54723 |
| ssc-miR-18a | THBS1, HIF1A | -1.54357 |
| ssc-miR-191 | NDST1, IL1A | -1.95017 |
| ssc-miR-193a-5p | TP73, ACVR1 | -4.36901 |
| ssc-miR-205 | LYN | -2.57918 |
| ssc-miR-221-3p | FOS, TICAM1, SELE | 2.502738 |
| ssc-miR-2320-5p | IL13, ITGB2, ADORA1, CD97, IL23R, CD180 | -2.21697 |
| ssc-miR-23a | IL6R | -2.87366 |
| ssc-miR-27b-3p | IL6R | -2.63737 |
| ssc-miR-30a-3p | IL1B, CCR7 | -2.14194 |
| ssc-miR-30a-5p | CD44, THBS1, MAP2K3 | -2.41906 |
| ssc-miR-30b-3p | THBS1, CCL16 | -2.36214 |
| ssc-miR-320 | TAC1, TFRC | -2.58277 |
| ssc-miR-328 | CD44, CFH | -2.75429 |
| ssc-miR-365-3p | ACVR1 | -4.32348 |
| ssc-miR-365-5p | BLNK | -4.89458 |
| ssc-miR-424-3p | FOS | -6.92963 |
| ssc-miR-425-3p | FN1 | -1.57611 |
| ssc-miR-4331 | SERPING1 | 2.490371 |
| ssc-miR-4334-3p | CCR7 | 2.222987 |
| ssc-miR-450b-3p | TLR9 | -2.43253 |
| ssc-miR-486 | CD40 | 1.91105 |
| ssc-miR-505 | CFHR1, CFH | -3.63539 |
| ssc-miR-542-5p | LY86 | -4.90563 |
| ssc-miR-574 | IL6, RXRA | -3.98769 |
| ssc-miR-7 | RELA | 3.066091 |
| Table S6. Cont. |  |  |
| miRNA name | Target genes | Fold change |
| ssc-miR-744 | CD44 | -3.84751 |
| ssc-miR-9-1 | NFKB1 | 2.983152 |
| ssc-miR-9-2 | NFKB1 | 2.983152 |
| ssc-mir-125b | CLU, AKT1, CASP6, STAT3, BMPR1B | -2.45236 |

Table S7. Summary of DE miRNAs whose targets are associated with immune responses on PID 7.

| miRNA name | Target genes | Fold change |
| --- | --- | --- |
| ssc-miR-1 | CEBPA, LRRC8A, SOX6, HDAC4, NOTCH2, G6PD, ASH2L, HSPD1 | -2.39392 |
| ssc-miR-146b | KIT, TRAF6 | -1.62421 |
| ssc-miR-193a-5p | MLL | -1.58147 |
| ssc-miR-196b-5p | HOXB7, HOXA9 | 2.140662 |
| ssc-miR-221-3p | TP53, KIT | 1.869522 |
| ssc-miR-365-5p | BCL11B, BLNK | -1.99331 |
| ssc-miR-450b-5p | BCL11B | -1.83442 |
| ssc-miR-505 | CACNB4 | -1.51547 |
| ssc-miR-542-5p | SYK | -1.9003 |
| ssc-miR-9-1 | BCL6 | 1.944785 |
| ssc-miR-9-2 | BCL6 | 1.944785 |

Table S8. Summary of differentially expressed miRNAs whose targets are associated with inflammatory responses on PID 7.

| miRNA name | Target genes | Fold change |
| --- | --- | --- |
| ssc-miR-1 | HDAC4, THBS1 | -2.39392 |
| ssc-miR-146b | NFKB1 | -1.62421 |
| ssc-miR-193a-5p | TP73, ACVR1 | -1.58147 |
| ssc-miR-221-3p | FOS, TICAM1, SELE | 1.869522 |
| ssc-miR-365-5p | BLNK | -1.99331 |
| ssc-miR-424-3p | FOS | -2.05524 |
| ssc-miR-505 | CFHR1, CFH | -1.51547 |
| ssc-miR-9-1 | NFKB1 | 1.944785 |
| ssc-miR-9-2 | NFKB1 | 1.944785 |


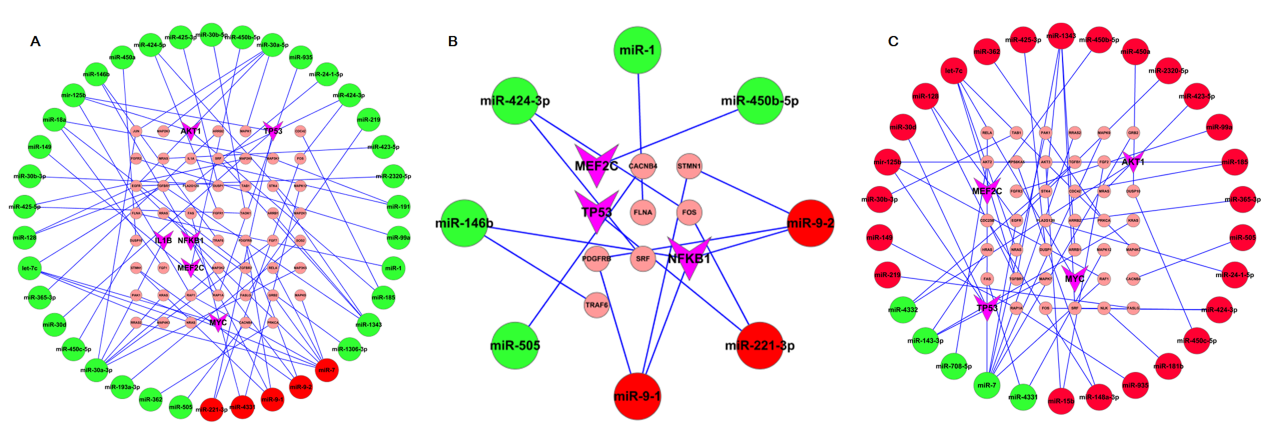


**Figure S1. The MAPK signaling pathway mediated by DE miRNAs between samples infected with H1N1 SwIV at different time points.** (A) The MAPK signaling pathway mediated by DE miRNAs between PID 4 and PID 0. (B) The MAPK signaling pathway mediated by DE miRNAs between PID 7 and PID 0. (C) The MAPK signaling pathway mediated by DE miRNAs between PID 7 and PID 4. In all pictures, larger red circles indicate up-regulated miRNAs while green ones indicate down-regulated miRNAs. Smaller pink circles indicate targets involved in the MAPK signaling pathway. Pink V symbols indicate target genes coding TFs.

**
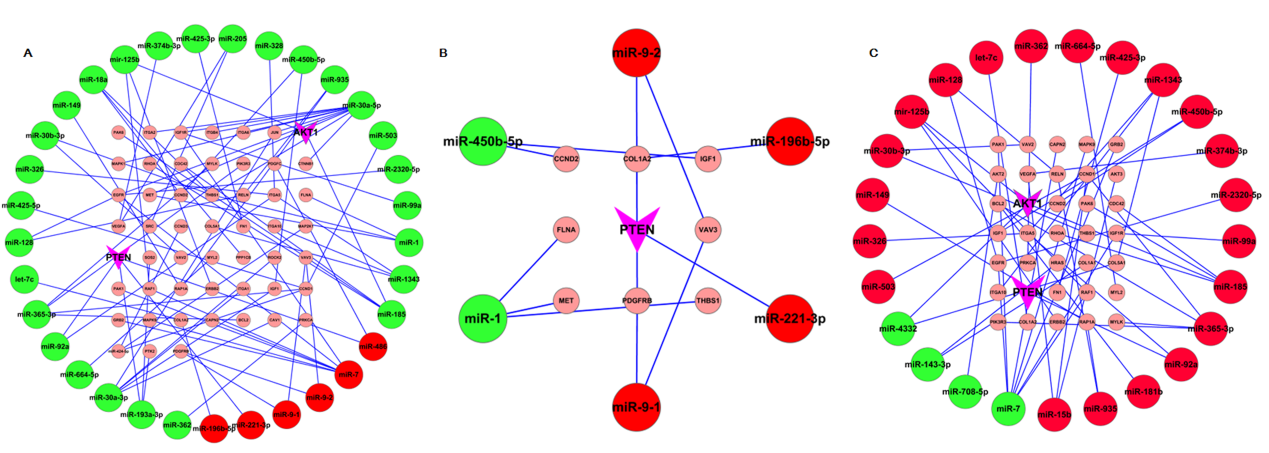
**

**Figure S2. The focal adhesion signaling pathway mediated by DE miRNAs between samples infected with H1N1 SwIV at different time points.** (A) The focal adhesion signaling pathway mediated by DE miRNAs between PID 4 and PID 0. (B) The focal adhesion signaling pathway mediated by DE miRNAs between PID 7 and PID 0. (C) The focal adhesion signaling pathway mediated by DE miRNAs between PID 7 and PID 4. In all pictures, larger red circles indicate up-regulated miRNAs while green ones indicate down-regulated miRNAs. Smaller pink circles indicate targets involved in the focal adhesion signaling pathway. Pink V symbols indicate target genes coding TFs.


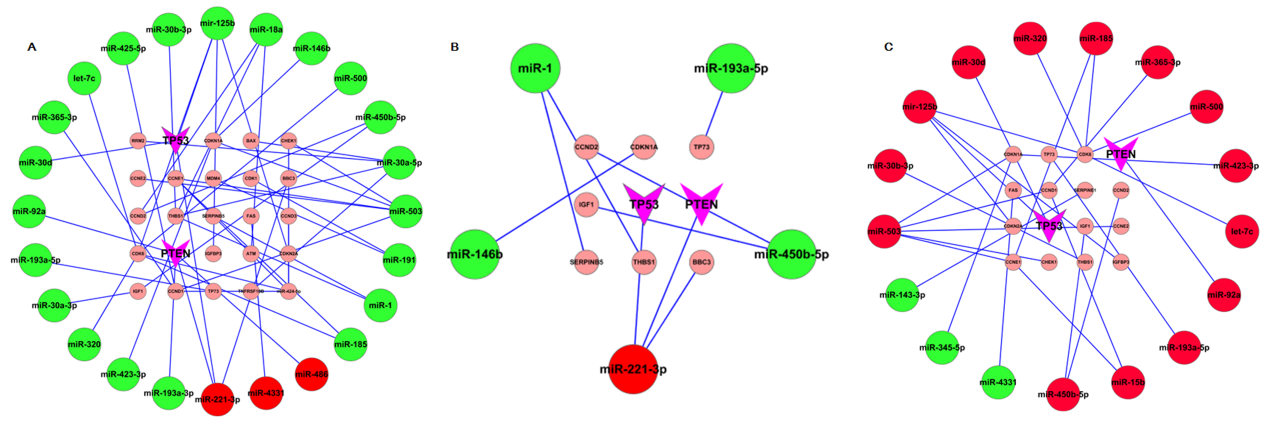


**Figure S3. The p53 signaling pathway mediated by DE miRNAs between samples infected with H1N1 SwIV at different time points.** (A) The p53 signaling pathway mediated by DE miRNAs between PID 4 and PID 0. (B) The p53 signaling pathway mediated by DE miRNAs between PID 7 and PID 0. (C) The p53 signaling pathway mediated by DE miRNAs between PID 7 and PID 4. In all pictures, larger red circles indicate up-regulated miRNAs while green ones indicate down-regulated miRNAs. Smaller pink circles indicate targets involved in the p53 signaling pathway. Pink V symbols indicate target genes coding TFs.


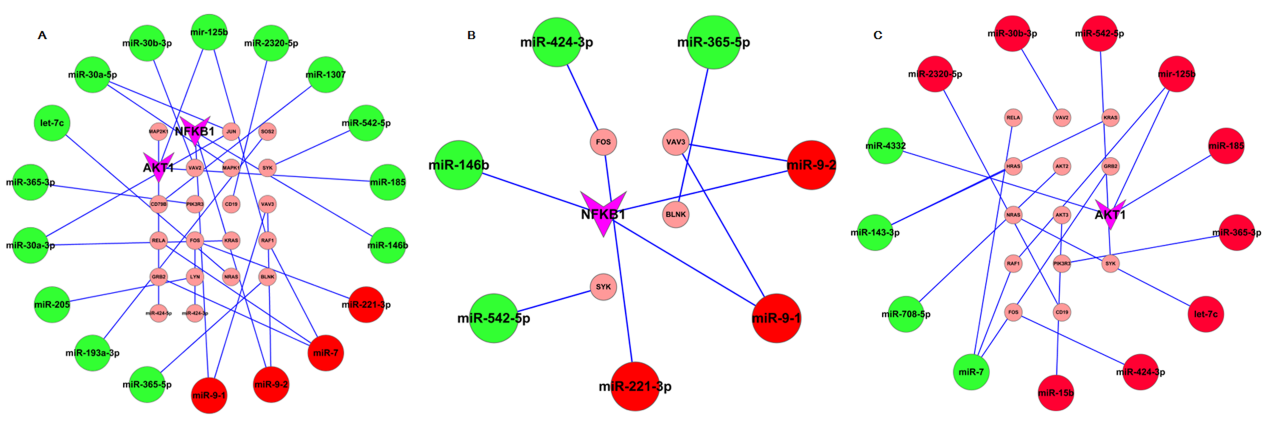


**Figure S4. The B cell receptor signaling pathway mediated by DE miRNAs between samples infected with H1N1 SwIV at different time points.** (A) The B cell receptor signaling pathway mediated by DE miRNAs between PID 4 and PID 0. (B) The B cell receptor signaling pathway mediated by DE miRNAs between PID 7 and PID 0. (C) The B cell receptor signaling pathway mediated by DE miRNAs between PID 7 and PID 4. In all pictures, larger red circles indicate up-regulated miRNAs while green ones indicate down-regulated miRNAs. Smaller pink circles indicate targets involved in the B cell receptor signaling pathway. Pink V symbols indicate target genes coding TFs.
